# Therapeutic Potential of Quercetin to Alleviate Endothelial Dysfunction in Age-Related Cardiovascular Diseases

**DOI:** 10.3389/fcvm.2021.658400

**Published:** 2021-03-30

**Authors:** Olina Dagher, Pauline Mury, Nathalie Thorin-Trescases, Pierre Emmanuel Noly, Eric Thorin, Michel Carrier

**Affiliations:** ^1^Department of Cardiac Sciences, Cumming School of Medicine, University of Calgary, Calgary, AB, Canada; ^2^Department of Surgery, Faculty of Medicine, Université de Montréal, Montreal, QC, Canada; ^3^Center for Research, Montreal Heart Institute, Montreal, QC, Canada

**Keywords:** endothelial (dys)function, flavonoids, quercetin, hypertension, atherosclerosis, senescence, aging, ischemia-reperfusion

## Abstract

The vascular endothelium occupies a catalog of functions that contribute to the homeostasis of the cardiovascular system. It is a physically active barrier between circulating blood and tissue, a regulator of the vascular tone, a biochemical processor and a modulator of coagulation, inflammation, and immunity. Given these essential roles, it comes to no surprise that endothelial dysfunction is prodromal to chronic age-related diseases of the heart and arteries, globally termed cardiovascular diseases (CVD). An example would be ischemic heart disease (IHD), which is the main cause of death from CVD. We have made phenomenal advances in treating CVD, but the aging endothelium, as it senesces, always seems to out-run the benefits of medical and surgical therapies. Remarkably, many epidemiological studies have detected a correlation between a flavonoid-rich diet and a lower incidence of mortality from CVD. Quercetin, a member of the flavonoid class, is a natural compound ubiquitously found in various food sources such as fruits, vegetables, seeds, nuts, and wine. It has been reported to have a wide range of health promoting effects and has gained significant attention over the years. A growing body of evidence suggests quercetin could lower the risk of IHD by mitigating endothelial dysfunction and its risk factors, such as hypertension, atherosclerosis, accumulation of senescent endothelial cells, and endothelial-mesenchymal transition (EndoMT). In this review, we will explore these pathophysiological cascades and their interrelation with endothelial dysfunction. We will then present the scientific evidence to quercetin's anti-atherosclerotic, anti-hypertensive, senolytic, and anti-EndoMT effects. Finally, we will discuss the prospect for its clinical use in alleviating myocardial ischemic injuries in IHD.

## Introduction

It is impressive to think that one single layer of cells tightly regulates homeostasis of the cardiovascular system. With its enormous surface area and its key location at the interface between circulating blood and tissue, the vascular endothelium has multiple physiological functions, such as modulation of the vascular tone and local regulation of coagulative, immune and inflammatory stimuli, in addition to providing a semipermeable barrier (1). A normally functioning endothelium appropriately arbitrates between opposing states of vasodilatation and constriction, permeability and non-permeability, adhesion and non-adhesion, as well as anti-thrombotic and pro-thrombotic conditions (1). Therefore, it is intuitive to imagine that a distortion in this equilibrium can result in adverse effects (2). Indeed, many cardiovascular diseases (CVD) are either a direct or indirect result of a dysfunction of the endothelium that fails to maintain moment-to-moment homeostasis, ultimately creating maladaptation in meeting organ metabolic demand and chronic damages (3). An example would be ischemic heart disease (IHD), which is the main cause of death from CVD (1). IHD itself represents an umbrella term for a group of clinical syndromes characterized by myocardial ischemia such as stable angina and acute coronary syndromes. Risk factors for endothelial dysfunction, and, by extension, IHD, include smoking, obesity, insulin resistance, diabetes, hypercholesterolemia, and physical inactivity (4). Phenomenal advances in pharmacology have enabled us to therapeutically target many of these risk factors, resulting in a significant decline in cardiovascular mortality over the last four decades (5). However, the use of drugs remains hampered by their toxicity, patients' tolerance and the limits of their clinical efficacy. In addition, endothelial dysfunction inevitably occurs with normal aging, fuelled by a process of irreversible cell cycle arrest termed senescence (6). For these reasons, there has been a burgeoning interest in introducing complementary therapies, such as dietary components, in the prevention of CVD (7).

Among promising nutraceuticals, a group of naturally occurring compounds found in plants, called flavonoids, have become increasingly popular. As early as in the 1990s, data from epidemiological studies have established a connection between a higher intake of flavonoid rich diets and a lower incidence of CVD (8). Quercetin has been singled out among flavonoids mainly because of its ubiquitous presence in our diets. It was also the first flavonoid to be discovered, precisely in the context of a vascular pathology. Indeed, in 1936, Albert Szent-Gyorgyi and his collaborators published the case of a patient who recovered from a bleeding disorder after receiving an infusion of a substance extracted from a Hungarian red pepper, which they called vitamin P, for “permeability” (9). Quercetin has since gained significant attention for its wide range of biological activities, some of which can mediate cardioprotective effects (10). In this review, we will examine quercetin's potential to alleviate CVD by protecting endothelial function. We will focus on three core pathophysiological mechanisms: atherosclerosis, hypertension and endothelial senescence. We will also cover quercetin's effects against endothelial-mesenchymal transition (EndoMT), as an additional, yet poorly explored, therapeutic avenue. Finally, we will discuss its potential use in secondary and tertiary prevention of endothelial dysfunction by taking the example of myocardial ischemic injury in IHD.

## Endothelial Dysfunction as a Target for Preventing Cardiovascular Diseases

Conceptually, the core feature of endothelial dysfunction is a disrupted nitric oxide (NO) bioavailability as a consequence of a reduced production by endothelial NO synthase (eNOS) from L-arginine and in favor of free-radicals generation (11). Different causal paths have been implicated, including shear stress, dyslipidemia, hyperglycemia, insulin resistance, hyperhomocysteinemia and, more recently, senescence and EndoMT. The mechanisms by which they can lead to endothelial dysfunction and CVD pathogenesis are broad and complex. Most often, many of these factors accumulate in one person where they cross talk and synergistically enhance dysfunction of the arterial wall. Treatment of these cardiovascular risk factors was shown to reverse endothelial dysfunction and simultaneously improve the incidence of cardiac events (12). Here, we will focus our attention on the mechanistic connections between hypertension, atherosclerosis, senescence, and endothelial dysfunction.

### Endothelial Dysfunction in Hypertension and Atherosclerosis

Endothelial dysfunction is seen as an early step in the development of hypertension and atherosclerosis (13, 14). Indeed, the functional characteristics of endothelial dysfunction include an impairment of endothelium-dependent vasodilation and endothelial activation marked by pro-inflammatory, proliferative, and procoagulatory states (14).

Upon activation, endothelial cells switch from a predominant NO signaling to an oxidative stress signaling mediated by reactive oxygen species (ROS) (15). While NO promotes inhibition of pro-inflammatory cytokine secretion, thrombosis, smooth muscle cell proliferation and immune cell extravasation, ROS induce nuclear transcription factor kappa B (NFκB) signaling, the main regulator of inflammation (15). In addition, the diseased endothelium acquires a pro-inflammatory state and becomes more permeable, allowing the avid accumulation of oxidized low-density lipoproteins (ox-LDLs) and macrophages in the subintimal layer, culminating in foam cell formation and fatty streaks which are hallmarks of atherosclerosis development (15). On the other hand, a defective L-arginine/NO pathway, impaired responsiveness to exogenous NO and reduced generation of platelet NO result in a state of predominant vasoconstriction and higher resting blood pressure (14). Furthermore, atherosclerotic lesions develop preferentially at arterial bifurcations, branching points and vessel curvatures, where the blood flow is disturbed (16). This suggests the importance of hemodynamic forces and mechanical stress, hence of hypertension, in the initiation of atherosclerosis. When considering the role of atherosclerosis in hypertension, a number of studies reported that atherosclerotic segments were accompanied by an altered function of eNOS in which it produces superoxide instead of NO (17). NADPH oxidase (NOX), which is induced by ox-LDLs, was shown to lie upstream to this eNOS alteration (17). Referred to as “eNOS uncoupling,” this oxidative pathway is also present in aged microvessels (18). It goes without saying that oxidative stress plays a critical role in endothelial dysfunction, and, as we will next, in stress-induced senescence.

This interconnection between endothelial dysfunction, atherosclerosis and hypertension has been confirmed clinically: using arterial dilatation as a non-invasive measure for assessing endothelial function, endothelial dysfunction has been documented in both hypertensive and atherosclerotic patients (12, 19–22). Using acetylcholine to induce endothelium-dependent dilation, a reduction in arterial dilation was observed in the forearm and coronary beds of patients with essential hypertension (12). Furthermore, the response to acetylcholine and adenosine was significantly decreased in patients with hypertension and left ventricular hypertrophy, indicating an impairment in both endothelium-dependent and endothelium-independent vasodilation (19). Ludmer et al. provided the first evidence of compromised endothelium-dependent vasodilation in the presence of atherosclerosis in humans (20). Using the acetylcholine test, they reported a paradoxical constriction in the coronary arteries of patients with both mild and advanced coronary artery disease (20). Endothelial dysfunction was also present in the vasculature of patients with coronary risk factors but no angiographic or ultrasound evidence of structural coronary artery disease (21). These studies suggest that endothelial dysfunction is detectable from the early stages of atherosclerosis and that it might even be a trigger mechanism (22).

Now endothelial dysfunction can be extended beyond the concept of a damaged conduit vessel to that of a defective vascular wall composed of layers of cells that are prone to aging. If endothelial dysfunction is the *primum movens* of hypertension and atherosclerosis, an upstream connection between the three could be linked to senescence.

### Senescence: The Natural Fate of Aging Cells

Successive replication (23) and harmful stimuli such as DNA damage, oxidative stress, and induction of mitochondrial dysfunction eventually impose a state of permanent proliferative arrest on cells (24, 25). This phenomenon, termed “senescence,” is well-recognized as one of the nine hallmarks of aging (26).

Despite being in cell cycle arrest, senescent cells (SCs) undergo profound phenotypic changes and remain metabolically active. In response to stress, they secrete a set of proteins collectively termed the senescence-associated secretory phenotype (SASP) (27, 28). These include pro-inflammatory cytokines (interleukin (IL)-6, IL-8, membrane cofactor proteins (MCPs) and macrophage inflammatory proteins) and chemokines, immune modulators, growth factors [hepatocyte growth factor, fibroblast growth factors, granulocyte-macrophage colony-stimulating factor, or transforming growth factor beta (TGF-β)] and hundreds of signaling molecules such as damage-associated molecular patterns, proteases, extracellular matrix (ECM) components [matrix metalloproteinases (MMPs)], serine/cysteine proteinase inhibitors (SERPINs), tissue inhibitor of metalloproteinases and cathepsins), proteases, bradykinins, and hemostatic factors (27–29). Without a doubt, the SASP plays an essential role in normal tissue development (30), wound healing (31), and cardiac repair (32). Transient expression of SASP during the acute phase of a tissue injury assists with repair and remodeling by recruiting the immune system to clear damaged cells and by stimulating progenitor cells to repopulate the damaged tissue (33). However, senescence becomes a double-edged phenomenon when ineffective clearance of SCs prolongs their residency. In the concept of “inflamm-aging,” aberrant focal accumulation of SCs creates a pro-inflammatory environment favorable for the onset of various pathological conditions, including endothelial dysfunction (34). Indeed, a growing body of evidence shows that SCs are prominent in diseased vascular walls (35), including in intact arteries from IHD patients (36, 37). Furthermore, although vascular cells have a finite replicative capacity, a combination of both damage-dependent replicative senescence and stress-induced senescence might be especially relevant to premature vascular aging and endothelial dysfunction (3, 38).

### The Vicious Circle of Endothelial Dysfunction

**Figure 1:**
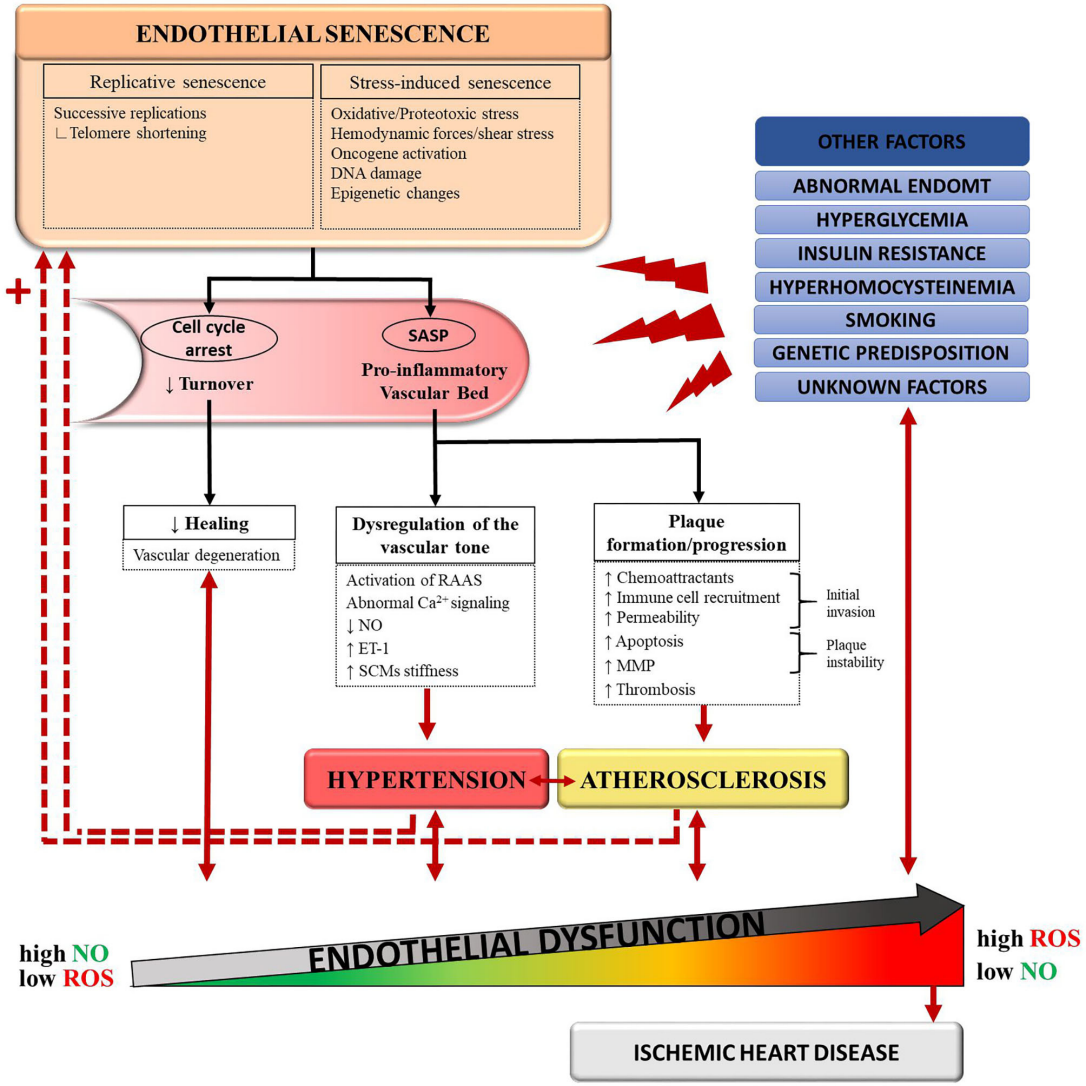
Schematic representation of the proposed connections between senescence, hypertension, atherosclerosis, and endothelial dysfunction. Normal aging and deleterious stimuli induce senescence in endothelial cells (ECs), vascular smooth muscle cells (VSMCs), and foam cells. Accumulation of these senescent cells favors a pro-inflammatory state of the vascular bed through the senescence-associated secretory pathway (SASP). In turn, the SASP promotes pathological changes leading to the development of hypertension and atherosclerosis. In a feedback manner, hypertension and atherosclerosis induce more stressors to an already dysfunctional and senescent vessel wall. This vicious circle translates into endothelial dysfunction and, eventually, ischemic heart disease. Other causal pathways of endothelial dysfunction include hyperglycemia, insulin resistance, abnormal endothelial-to-mesenchymal transition (EndoMT), genetic predisposition and detrimental lifestyle habits such as smoking. ET-1, endothelin-1; MMP, matrix metalloproteases; NO, nitric-oxide; RAAS, renin–angiotensin–aldosterone system; ROS, reactive oxygen species.

Senescence of a vascular wall leads to two immediate consequences: induction of a pro-inflammatory environment by the SASP and a reduction in the turnover of vascular cells (Figure 1). In atherogenesis, plaque initiation could be driven by senescent endothelial cells through their increased secretion of chemoattractant factors and adhesion molecules, which allow for the initial invasion of circulating monocytes into the vessel wall (39). Conversely, clearance of senescent vascular cells lowered the pathogenesis of atherosclerosis in a mouse model of severe dyslipidemia (36). In addition, a senescent endothelium presents an altered cellular lining, causing a break in selective permeability (40). This can facilitate migration of ox-LDLs to the subendothelial layers. The SASP can also stimulate vascular smooth muscle cells to secrete elastase and MMPs, which can digest components of the extracellular matrix (41). An amplified degradation of the extracellular matrix could create a rupture-prone vulnerable plaque. Thus, senescence of vascular cells leads to vascular inflammation and plaque progression. This vascular inflammation also raises the possibility of a multistep role of senescence in hypertension, although the link between the two is less clearly established. The SASP could play a role in the dysregulation of the vascular tone: as an example, it was found to activate the renin–angiotensin–aldosterone system (35).

With aging, the clearance of SCs by the immune system is decreased, contributing to the accumulation of SCs (33, 42). Senescence therefore begets senescence (Figure 1), a phenomenon that has been validated in mice (43). As senescence induces more senescence, the processes involved in the pathogenesis of atherosclerosis and hypertension are further amplified. In parallel, important changes in the extra-cellular matrix (ECM) protein composition occur with aging and promote arterial stiffening (44). A rigid arterial wall causes systolic hypertension, which in turn, contributes to atherosclerosis through shear stress (3). As mentioned before, other cardiovascular risk factors often coexist, such as metabolic disturbances, obesity, smoking or genetic predisposition, accelerating this deleterious process (12). The vascular wall eventually gets caught into a vicious circle where it must face more stressors with less protective capacities (3). Therefore, it is possible to acknowledge a cyclical, rather than sequential, relationship between senescence, hypertension and atherosclerosis, all contributing to endothelial dysfunction. In the next sections, we will explore quercetin's potential to target this triad of endothelial dysfunction.

## Quercetin

### Classification and Structure

**Figure 2:**
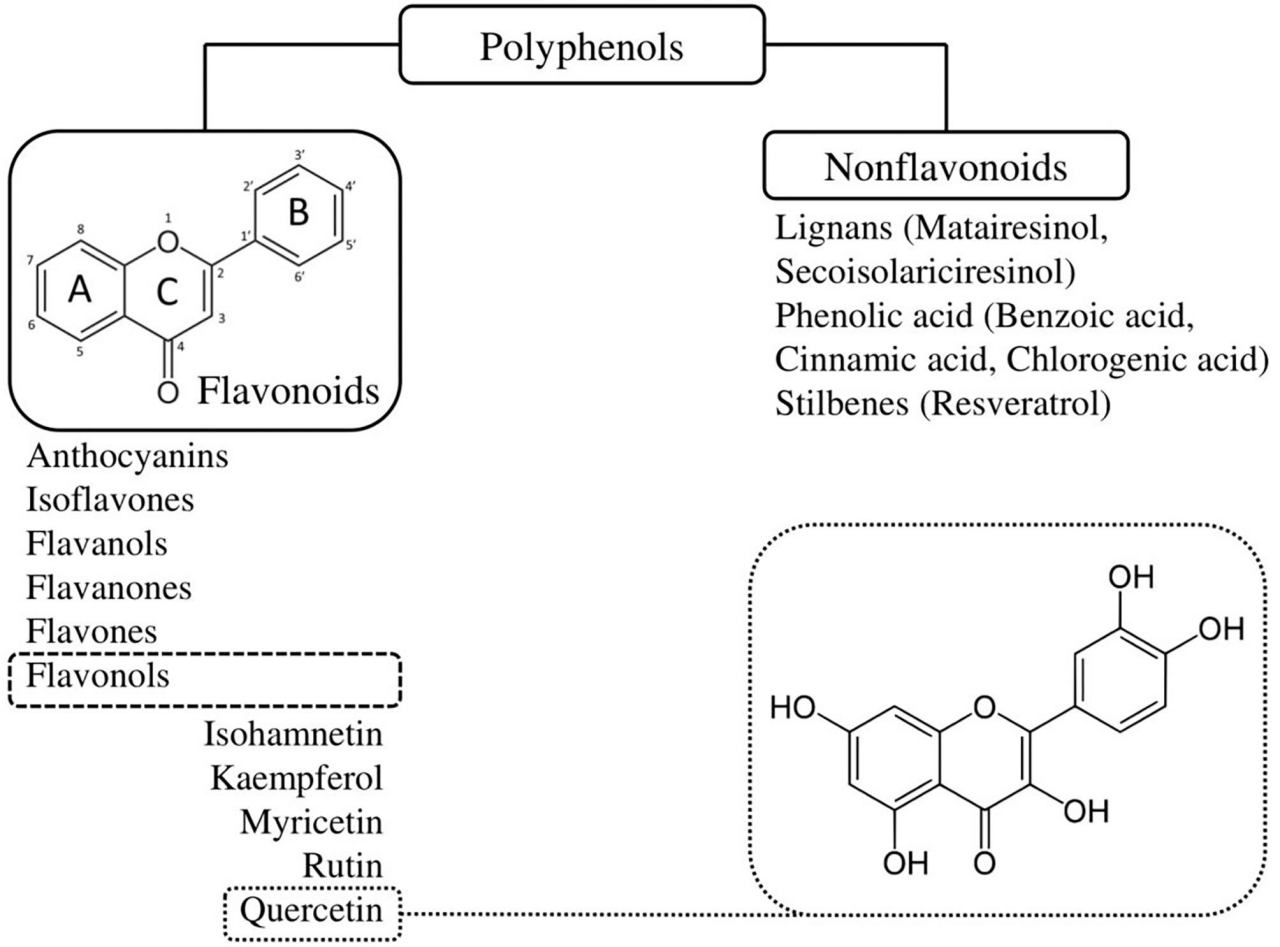
Classification and chemical structure of quercetin, a family member of flavonoids. Quercetin is a pentahydroxyflavone, having five hydroxyl groups placed at the 3-, 3'-, 4'-, 5-, and 7-positions. Combined with the pyrocatechol, a benzene ring, this chemical structure allows them to act as radical scavengers, explaining, in part, quercetin's strong antioxidant properties.

Quercetin is part of a larger family of molecular compounds, named flavonoids, which share a common hydroxylated 3-ringed skeleton with attached hydroxyl groups (45) (Figure 2). Combined with the pyrocatechol, a benzene ring, this chemical structure allows them to act as radical scavengers, explaining, in part, flavonoids' antioxidant property (45). Flavonoids are themselves part of a large class of plant-derived substances named polyphenols (46). Flavonoids include several subclasses such as flavonols, flavones, flavanols, flavanones, isoflavones, and anthocyanins (46). They exist in most of the plants and play a variety of biological activities involved in vegetative growth (46). Being phytochemicals, flavonoids cannot be synthesized by humans or animals, but they are ubiquitously present in our diet (46). They are found in virtually all fruits and vegetables, as well as in seeds, nuts, tea and red wine (46). The mean daily intake of flavonoids in Australian, European and US adult populations has been estimated at 435 mg/day (47).

Structurally, quercetin is not only found in its free (aglycone) form, but also in various conjugated forms with glycosides or methyl ethers attached to the hydroxyl groups. Glycosylation preferentially occurs at the 3-hydroxyl position, such as quercetin 3-O-β-D-glucoside (isoquercetrin) or quercetin 3-O-galactoside (hyperoside), whereas methylation usually occurs at the 3', 4', or 7-hydroxyl positions, such as 3-methylquercetin (isorhamnetin) (48). Some quercetin derivatives even contain both glycosyl and ethyl groups. For example, tamarixetin has a glucose residue at the 3' position and a methyl group at the 4' position (48). Extensive studies of the biological activities of quercetin have shown that the various derivatives have different levels of efficacy. For example, free quercetin was found to have the strongest antioxidant activity, confirming the important contribution of unbound hydroxyl groups (49). Among its metabolites, free quercetin was also the most effective recombinant human angiotensin-converting enzyme (ACE) 2 inhibitor (50). Tamarixetin and isorhamnetin demonstrated a stronger inhibition of lipid peroxidation compared to quercetin (49, 51). Tamarixetin also exhibited the highest anti-inflammatory activity, suggesting that unlike the antioxidant activity, anti-inflammatory activity is not correlated with the number of free hydroxyl groups (49). These disparities in biological activities prompt the synthesis of particular metabolites that present the highest efficacy of a desired effect. This can be achieved by inducing glycosylation or methylation using purified biocatalysts *in vitro* and native or metabolically engineered microorganisms (48).

### Bioavailability and Pharmacology

The chemical structure of aglycone quercetin makes it hydrophobic (52). Its solubility in water is 2.1 mg/L at 25°C, while it is up to 2 g/L in ethanol (52). This physical property limits its absorption and practical use in preparation forms as a dietary supplement. Initial investigations on the pharmacokinetics of quercetin in humans suggested very poor oral bioavailability after a single oral dose (~2%) (53). The absorption was found to increase to 3–17% when quercetin was consumed in a glycosidic bond compared to its aglycone form (53). Different delivery systems using nanotechnology have since been developed to further improve its water solubility and bioavailability, for example, by binding it to solid lipid carriers or nanosized polymeric micelles (54). A pharmacokinetic study in beagle dogs showed that quercetin encapsulated in polymeric micelles induces a 2.19-fold longer half-life and a relative oral bioavailability increased by 286% as compared to free quercetin (55).

Since dietary quercetin is usually present in its glycosylated form, it can be rapidly hydrolyzed by β-glucosidases in the digestive tract, which makes it easier for absorption by the colonic mucosa (56). It is then transferred to the liver through the portal circulation where it undergoes first-pass metabolism and is almost completely metabolized by glucuronidation, methylation, or sulfonylation (56, 57). Peak plasma concentration following an oral quercetin dose is reached anywhere from 0.6 to 4 h (58–60). Quercetin glucuronides are the main circulating metabolites and are rapidly eliminated in the urines (57, 60). This short elimination half-life is another limit to quercetin's medical use. Furthermore, quercetin's metabolism seems to be dependent on individual characteristics. A correlation between β-glucuronidase activity and the apolipoprotein (apo) E phenotype may explain the efficacy of quercetin in patients with apoE3 phenotype as opposed to those expressing apoE4 (61, 62). On the other hand, an increased expression of β-glucuronidase was correlated with inflammation, raising the hypothesis that quercetin may be more effective under inflammatory conditions (63). This is especially favorable as endothelial dysfunction is often associated with a pro-inflammatory state.

When it comes to pharmacokinetic interactions, conclusions are still open to debate. Some studies investigated the effects of quercetin on the cytochrome P450 system and have noted a potential inhibitory effect of quercetin on the activity of selected enzymes (64). Studies in pigs have shown that quercetin can decrease bioavailability of cyclosporine and increase that of digoxin, verapamil and various chemotherapeutic agents (65–67). However, conflicting results between *in vitro* and *in vivo* studies have been found (67). Quercetin was also reported to bind to DNA gyrase enzyme in bacteria, which could competitively inhibit fluoroquinolone antibiotics' activity (68). One case report of a clinically relevant warfarin interaction resulting in supratherapeutic international normalized ratio values has been documented in an elderly patient who ate large quantities of scuppernongs, a quercetin-containing muscadine grape (69).

### Safety Profile

In the 1970s, *in vitro* mutagenicity of quercetin in the Ames test was reported, leading to concerns about its safety (70). Later, *in vivo* studies contradicted these findings and showed that quercetin may be protective against carcinogens (70). Since 1999, it is classified as a group 3 agent (“not classifiable as to its carcinogenicity” to humans) by the International Agency for Research on Cancer (70). In 2010, QU995, a highly pure form of quercetin, was granted a “generally recognized as safe” (GRAS) status by the U.S. Food and Drug Administration (59). Many other quercetin formulas have since been developed and made widely available over the counter as oral dietary supplements or added ingredient to numerous multivitamin preparations.

Quercetin is generally well-tolerated. Some minor side-effects such as mild headache, nausea, and tingling of the extremities were observed in long-term supplementation at 1,000 mg/day (67). In Canada, the recommended maximum daily dose is 1,200 mg (67). A therapy as long as 12 weeks showed no evidence of toxicity, but data on long-term safety are lacking (67). Nephrotoxicity has been reported with high intravenous doses in cancer patients (67). Quercetin was not found to cause critical adverse effects on fetal growth in rats, but human studies are not available (67). Therefore, dosages above those found in foods should be avoided by pregnant women and nursing mothers (67).

### A Recent Resurgence in Interest

While it was previously known as “vitamin P,” the National Nutrition Institute withdrew its status in 1950 when it was found to be a non-essential nutrient (71). Added to a mislabeling of genotoxicity, altogether this contributed to a loss of interest in the molecule. In 1993, however, the Zutphen Elderly Study first reported a 50% reduction of mortality from IHD in Dutch men who consumed >29 mg flavonoids/day compared with those who consumed <19 mg (72). Around the same time, the concept of the French paradox emerged from the contradictory observation of a low IHD-related mortality despite high intakes of dietary saturated fat among the French population (73). Most debates have focused on high consumption of red wine, which contains a variety of polyphenols, including flavonoids (73). Other epidemiological studies soon followed and showed a positive correlation between dietary intake of flavonoids and a reduced incidence of stroke, myocardial infarction and mortality from IHD (74).

**Figure 3:**
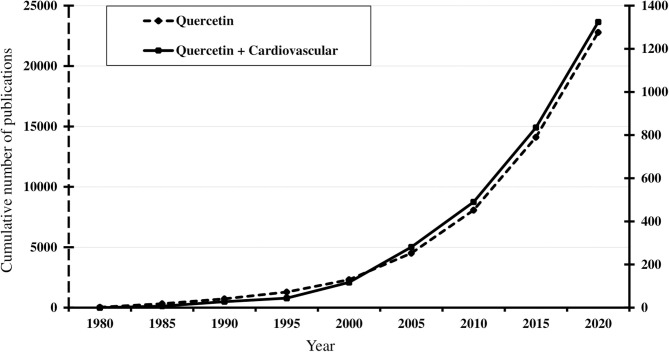
Timeline of the cumulative number of published results, from 1980 to 2020, of an online PubMed literature search using “Quercetin” (dotted line) and “Quercetin [and] Cardiovascular” (solid line) as the search term. Note the progressive increase from the mid-1990s, coinciding with publication of observational studies associating flavonoid consumption with lower cardiovascular risks. Search performed January 10, 2021 (www.ncbi.nlm.nih.gov/pubmed).

Over the years, quercetin was found to have a diverse array of biological properties, such as anti-inflammatory, anti-oxidative, anti-platelet, anti-diabetic, anti-histaminic, anti-carcinogenic, anti-bacterial, immunomodulating, and neuroprotective (75). These prominent effects have sparked attention and hope among the scientific community. As of the end of 2020, there are more than 20,000 published articles on quercetin, and this number exceeds 120,000 when including all flavonoids (Figure 3). Despite quercetin being discovered for its role in treating capillary wall dysfunction, it has gained more popularity in oncology and sports medicine, each counting 50% more publications than the field of cardiovascular research. However, its promising benefits for the endothelium cannot be ignored (Table 1).

**Table 1:** Summary of the main *in vitro* and *in vivo* cardiovascular effects of quercetin.

**Effects**	**Subjects**	**Evidence and possible mechanisms**	**References**
Anti-atherosclerotic	Animals	Reduced atherosclerotic plaque areas	(76–84)
		Increased concentration of SCFAs in the intestinal tract of ApoE^−/−^ mice	(85)
		Promoted cholesterol-to-bile acid conversion and cholesterol efflux	(78, 82, 86–92)
		Downregulated PCSK9 expression in RAW264.7 cells and in ApoE^−/−^ mice	(90–92)
		Normalized plasmatic/hepatic activities of HMG-CoA reductase in Wistar rats	(93)
		Downregulated MMP-1, MMP-2, MMP-9	(94–98)
		Decreased platelet aggregation in a concentration-dependent manner	(99)
		Inhibited thrombus formation through intracellular Ca^2+^ mobilization, granule secretion, and integrin activation	(100)
		Inhibited phosphorylation of signaling proteins downstream of glycoprotein VI	(100–102)
		Decreased ox-LDLs accumulation and foam cell formation	(92, 95, 103–106)
		Attenuated LDL oxidation	(107, 108)
		Decreased expression of adhesion molecules (ICAM-1, VCAM-1)	(109–111)
		Inhibited LOX-1 in RAW264.7 cells	(95)
		Decreased inflammatory cytokines, MCP-1 and COX-2 in RAW264.7 cells	(95)
	Humans	Reduced total and LDL-cholesterol in patients with metabolic syndrome traits^*^	(112)
		Reduced total and LDL-cholesterol in metabolically healthy patients^*^	(113)
		Decreased platelet aggregation in citrated whole blood in a concentration-dependent manner	(114)
		Increased cAMP levels, inhibition of ADP-induced platelet aggregation	(115, 116)
		Decreased expression of adhesion molecules (ICAM-1, VCAM-1)	(117, 118)
		Reduced plasma concentration of ox-LDLs	(119, 120)
Vasodilating	Animals	Improved Ach-induced relaxation of aortic rings harvested from hypertensive rats	(121)
		Reduced systolic, diastolic, and mean arterial blood pressure in hypertensive rats	(122–124)
		Improved endothelium-dependent aortic vasodilatation and eNOS activity	(125–129)
		Reduced eNOS uncoupling	(124, 130, 131)
		Inhibited LTCCs and enhanced VGKCs in coronary artery rings	(132)
		Reduced ACE activity in Wistar rats	(133)
	Humans	Decreased expression of ET-1 gene/protein, and production of ET-1	(134–136)
		Reduced systemic blood pressures in both normotensive and hypertensive patients^*^	(137–139)
Senolytic	Animals	Reduced viability of senescent HUVECs	(140)
		Combined with dasatinib, reduced the number of p16-positive SCs in fat and liver from old mice	(140)
		Combined with dasatinib, increased median lifespan in old mice	(43)
		Increased the density of Sirt1 in aorta of ApoE–/– mice	(141)
		Decreased expression of β-galactosidase and improved cell morphology of HAECs	(141)
	Humans	Decreased expression of AATK, CDKN2A, and IGFBP3 in HAECs	(141)
		Combined with dasatinib, reduced the number of adipose tissue SCs and circulating SASP factors	(43, 142)
Myocardial protectant	Animals	Alleviated ischemia-induced reduction in LVSP	(143–147)
		Reduced the decline in LVEF and FS induced by ischemia	(148)
		Reduced myocardial infarct size	(149–151)
		Lowered levels of CK, CK-MB, cTnT, and LDH post infarction	(144, 147–149, 151–155)
		Decreased leukocytes' infiltration and edema in infarcted myocardium	(149, 152, 153)
		Inhibited HMGB1 and TLR4 in cardiomyocytes	(151, 153)
		Up-regulated PPAR-γ positive myocardial cells	(148)
		Protected against calcium overload by downregulating calpain 1 and 2	(152)
	Humans	Reduced levels of IL-1β and TNF-α in patients with stable angina	(156)
		Improved profile of cardiac biomarkers and LVEF in patients with acute myocardial infarction	(157)

* *From a meta-analysis of randomized controlled trials. AATK, apoptosis-associated tyrosine kinase; ACE, angiotensin-converting enzyme; ADP, adenosine diphosphate; CDKN2A, p16, cyclin-dependent kinase inhibitor 2A; COX-2, cyclooxygenase-2; cAMP, cyclic adenosine monophosphate; CK, creatine kinase; CK-MB, creatine kinase-MB; cTnT, cardiac troponin T; eNOS, endothelial nitric oxide synthase; ET-1, endothelin-1; FS, fractional shortening; HAECs, human Aortic Endothelial Cells; HMGB1, high mobility group box protein 1; HUVECs, human umbilical vein endothelial cells; ICAM-1, intercellular adhesion molecule 1; IGFBP3, insulin-like growth factor binding protein-3; IL-1β, interleukin-1β; LDH, lactate dehydrogenase; LOX-1, lectin-like ox-LDL receptor-1; LTCCs, L-type Ca^2+^ channels; LVEF, left ventricular ejection fraction; LVSP, left ventricular systolic pressure; MCP-1, monocyte chemoattractant protein-1; MMPs, matrix metalloproteases; NO, nitric oxide; ox-LDLs, oxidized low density lipoproteins; PCSK9, proprotein convertase subtilisin/kexin type 9; PPAR-γ, peroxisome proliferator-activated receptor gamma; SCs, senescent cells; SCFAs, short-chain fatty acids; SASP, senescence-associated secretory phenotype; SIRT1, sirtuin-1; TLR4, Toll-like receptor 4; TNF-α, Tumor necrosis factor alpha; VCAM-1, vascular cell adhesion protein 1; VGKCs, voltage-gated K^+^ channel*.

## Cardiovascular Protective Properties of Quercetin

### Anti-Atherosclerotic Effects of Quercetin

With the increasing epidemic of the metabolic syndrome, the burden of atherosclerosis-related disorders persists despite the current pharmacologic treatment of dyslipidemia (64). Therefore, finding additional anti-atherogenic drugs remains a topic of interest (158). Considerable evidence from experimental data indicates that quercetin may protect against atherosclerosis by interfering with multiple pathways involved in disease progression (Table 1). Several high-fat animal models exhibited reduced atherosclerotic plaque areas when exposed to quercetin (76–84). This observation was associated with a prevention of atherosclerosis-related acute aortic syndromes: in a mouse model with an exaggerated degeneration of the elastic lamina: administration of quercetin 2 weeks before inducing aortic diseases was found to reduce the incidence of aneurysms, dissections and aortic ruptures (109).

First, quercetin could positively regulate the metabolism of lipids. A recent systematic and meta-analysis of 16 randomized controlled trials (RCTs) published between 2007 and 2017 looked at the effects of quercetin on lipid profiles of patients with metabolic syndrome traits (112). A pooled analysis revealed that quercetin leads to a significant reduction in total and LDL cholesterol, without affecting triglyceride levels (112). The daily doses and treatment durations used in the trials varied greatly, from 3.12 to 3,000 mg/ day and from 3 to 12 weeks (112). Another meta-analysis of 9 RCTs done in overweight and obese subjects found that quercetin supplementation significantly reduces LDL cholesterol levels at doses of ≥250 mg/day and for a total dose ≥14,000 mg (159). Similar findings were observed in metabolically healthy non-obese adults after an 8-week regimen, with comparable effects among men and women (113). Recent studies have highlighted the influence of the gut microbiota on host metabolic health through its metabolites, especially short-chain fatty acids, which have been linked with improved lipid metabolism (160, 161). Quercetin was shown to increase the concentration of short-chain fatty acids in the intestinal tract of ApoE knockout (ApoE^−/−^) mice (85). Experiments on both *in vivo* rodent models and murine cultured macrophages (RAW264.7 cells) have suggested that quercetin promotes cholesterol-to-bile acid conversion and cholesterol efflux by upregulating activity of hepatic *CYP7A1*, liver X receptor α, *ABCG1, ABCA1*, and *LDLR* (78, 82, 86–92). Quercetin also downregulated *PCSK9* expression in RAW264.7 cells and in ApoE^−/−^ mice (90–92). HMG-CoA reductase plays a major role in the regulation of cholesterol metabolism as a rate limiting enzyme in the pathway of cholesterol biosynthesis (162). Results relating the effects of quercetin on HMG-CoA reductase activity have been inconsistent (86, 93). Wistar rats fed with a diet containing 0.4% quercetin for 5 weeks did not express a change in the enzyme's activity (86). However, in a model of isoproterenol (ISO)-induced myocardial infarction in Wistar rats, a 2 week oral quercetin pre-treatment at a dose of 10 mg/kg normalized plasmatic and hepatic activities of HMG-CoA reductase (93). Another protective mechanism of quercetin involving enhancement of autophagy by aortic macrophages was highlighted in ApoE^−/−^ mice (84).

Second, quercetin has been suggested to downregulate the expression of MMP-1, MMP-2, and MMP-9 in studies using molecular modeling techniques, cultured endothelial cells, murine macrophage cells and in hypertensive rats, an effect that translates in the prevention of plaque instability (94–98).

Third, platelet aggregation at the site of an unstable plaque also contributes to acute complications of atherosclerosis. Quercetin was found to have an antiaggregatory effect on rat platelet-rich plasma in a concentration-dependent manner (99). This was also observed in human citrated whole blood: using samples from 100 healthy volunteers, the minimal antiaggregatory concentration of quercetin was estimated at 15.26 μM (114). A synergistic enhancement of antiplatelet effect was noted when quercetin was added to aspirin (100). The half maximal inhibitory concentrations (IC_50_) values for the inhibition of platelet aggregation decreased from 10.83 μM when using aspirin alone, to 3.32, 2.99, and 1.11 μM upon co-administration of 2.5, 5, and 10 μM quercetin, respectively (100). In addition, isorhamnetin and tamarixetin, two methylated metabolites of quercetin, were shown to inhibit platelet aggregation and thrombus formation *in vitro* through effects on activation processes such as intracellular Ca^2+^ mobilization, granule secretion, and integrin activation (100). Their antithrombotic effect was confirmed with laser-induced thrombi in mouse cremaster arterioles (100). In human platelets, quercetin significantly increases cyclic AMP levels and inhibits arachidonic acid and adenosine diphosphate (ADP)-induced platelet aggregation (115, 116). Antiplatelet effects of quercetin and its metabolites have also been associated to inhibition of the phosphorylation of signaling proteins downstream of glycoprotein VI, namely the Src family tyrosine kinases Fyn and Syk, the phospholipase Cγ2 and the linker for activation of T cells (100–102).

Fourth, once oxidized in the intima, LDLs transform into an antigenic factor, ox-LDLs, which attract monocyte-derived macrophages to the vascular wall, thereby initiating a phagocytic process leading to foam cell formation (163). Accumulation of foam cells is an early step in the pathogenesis of atherosclerosis (163). In their study, Kawai et al. used mAb14A2, a novel monoclonal antibody binding quercetin, to stain aortic samples in Japanese subjects (164). Their results revealed that quercetin metabolites accumulate in atherosclerotic lesions, but not in normal-appearing aorta (164). In addition, intense staining was primarily localized with foam cells, suggesting a potential cellular target of quercetin (164). Several studies done on cultured cells showed that quercetin can attenuate ox-LDLs accumulation, foam cell formation, as well as ox-LDLs induced cytotoxicity and calcification (92, 95, 103–106). Interestingly, quercetin significantly reduced plasma concentrations of ox-LDLs in two RCTs (119, 120). A retrospective comparison of the participants' apoE genotypes revealed no significant inter-group difference in the reduction of ox-LDLs between the apoE3 and apoE4 subgroups (62). This lowering effect on ox-LDLs might be achieved through direct attenuation of LDL oxidation: the lag time of LDL oxidation was increased by 3- to 4-fold after administration of quercetin *in vitro* and in rats (107, 108). The authors proposed two mechanisms contributing to this attenuation of LDL oxidation: inhibition of copper-induced LDL oxidation, as well as up-regulation of Paraoxonase 1 (PON1) and its protective capacity against LDL oxidation (107, 108). Lectin-like ox-LDL receptor-1 (LOX-1) is a scavenger receptor that mediates uptake of ox-LDLs by macrophages (165). Administration of anti-LOX-1 antibodies was shown to inhibit atherosclerosis by decreasing these cellular events (165). Quercetin was shown to block LOX-1 in RAW264.7 cells (95). Moreover, ox-LDLs activate endothelial cells by inducing cell adhesion molecules, especially vascular cell adhesion molecule-1 (VCAM-1) and intracellular cell adhesion molecule-1 (ICAM-1) (166). Quercetin was found to downregulate ICAM-1 expression in diabetic rats and human endothelial cells (110, 117). Quercetin and isoquercetin were shown to attenuate VCAM-1 expression in mice, HUVECs and rat intestinal microvascular endothelial cells by suppressing multiple pathways including caveolin-1 (CAV-1), Toll-like receptor 4 (TLR4) and NFκB (109, 111, 118). As previously mentioned, ox-LDLs also stimulate eNOS uncoupling and ROS overproduction by macrophages and endothelial cells *via* activation of NOX (167, 168). In ApoE^−/−^ mice, quercetin partially reversed NOX expression and inhibited ox-LDL induced ROS formation in macrophages (83).

Finally, atherosclerosis is also a chronic inflammatory disease mediated by a network of pro-inflammatory cytokines. Quercetin's administration was associated with a decrease in multiple inflammatory cytokines, such as IL-1α, IL-1β, IL-2, IL-10, TNF-α, macrophage chemoattractant protein-1 and cyclooxygenase-2 (95). The impacts of quercetin on such a wide range of inflammatory markers are in favor of a multi-target effect of signal transduction.

### Vasodilating Effects of Quercetin

Several *ex-vivo* reactivity studies have shown a vasodilating ability of quercetin in rat aorta, portal vein, mesenteric arteries and coronary arteries (169–171) (Table 1). In addition, Choi et al. reported that quercetin acutely improved acetylcholine-induced relaxation of aortic rings harvested from two-kidney, one-clip (2K1C) hypertensive rats (121).

Quercetin's BP lowering effects were first documented *in vivo* in spontaneously hypertensive rats (122). Rats exposed to quercetin had a significant lower systolic (−18%), diastolic (−23%), and mean (−21%) arterial BP (122). In normotensive rats, endothelial dysfunction induced by a high-fat high-sucrose diet was prevented by the supplementation with quercetin for 28 days: both endothelium-dependent aortic vasodilatation and eNOS activity were improved by quercetin (125). The vasomotor protective effects of quercetin were also demonstrated in mice exposed to lipopolysaccharide-induced endotoxemia. Whether given before or after lipopolysaccharide injection, quercetin dose-dependently restored eNOS expression while abolishing inducible NO synthase (iNOS) (126).

Several hypotheses have been formulated regarding the up regulation of eNOS activity induced by quercetin. Some authors have suggested that quercetin phosphorylates eNOS by an AMP-activated protein kinase-dependent mechanism (127). Li et al. observed, in bovine aortic endothelial cells, that quercetin induced phosphorylation of eNOS at serine 1179 in a concentration, time-dependent manner; this effect was abolished by H-89, an inhibitor of protein kinase A (128). Using the same primary cell cultures, Khoo et al. proposed that quercetin stimulates eNOS phosphorylation at serine 1179 by causing a rapid increase in intracellular Ca^2+^ (129).

Other calcium-mediated vasoactive effects of quercetin have been proposed. L-type Ca^2+^ channels (LTCCs) and voltage-gated K^+^ channels (VGKCs) play a tonic role in the regulation of arterial vasomotricity and are commonly expressed in vascular smooth muscle cells (172, 173). LTCCs are involved in excitation-contraction coupling while VGKCs are critical for restoring the resting membrane potential (172, 173). Large (big)-conductance Ca^2+^-sensitive potassium channels (BK) and VGKCs are closely associated with coronary arterial smooth muscle vasodilatation (174). Of note, aging is associated with a reduced expression of BK channels in coronary arteries, which is consistent with a higher frequency of spontaneous vasospasmic activity in elderly people (174). Hou et al. showed that quercetin can inhibit LTCCs and enhance VGKCs in rat coronary artery rings, resulting in a decrease of the vasocontractions induced by high-K^+^ depolarizing solution (132). Moreover, coronary vasodilation induced by quercetin was not lost after denuding the arterial rings of their endothelium, suggesting that quercetin can also promote its vasodilatory effect through VSMC-mediated mechanisms (132). Cogolludo et al. noted that quercetin could activate BK channels in coronary artery myocytes while generating hydrogen peroxide (H_2_O_2_) (175). Although H_2_O_2_ is considered as a relaxing endothelium-derived hyperpolarizing factor (176) and can also activate the soluble guanylate cyclase as does NO (177, 178), the data of Cogolludo et al. may nonetheless represent an instance where quercetin behaves as a pro-oxidant rather than a vasodilator.

Endothelin (ET) is one of the most potent vasoconstrictors and is mainly produced by the vascular endothelium (179). ET-1 plays a major role in the homeostasis of the cardiovascular system. ET-1 has been associated with increased oxidative stress and endothelial dysfunction in humans (179). It was shown to stimulate eNOS uncoupling, therefore superoxide production, and promote vasoconstriction *via* activation of NOX (130, 180). ET-1 can further reduce NO bioavailability by interfering with eNOS expression through protein kinase C (PKC)-mediated activation of STAT3 (181). These data indicate that diminished ET-1 concentrations may be accompanied by elevated NO bioavailability (181). Lodi et al. showed that quercetin significantly decreased expression of ET-1 in human umbilical artery smooth muscle cells and human vein endothelial cells (HUVECs) co-culture model exposed to TNF-α-induced change in vasomodulatory molecules (134). Zhao et al. also showed that quercetin decreases ET-1 production in thrombin-stimulated HUVECs in a concentration-dependent manner, with an IC_50_ of 1.54 μmol/L (135). In rat aortic rings, quercetin prevented ET-1-induced PKC activation, with a subsequent decrease in superoxide production (130). Moreover, chronic treatment with quercetin reduced blood pressure and improved endothelial function in deoxycorticosterone acetate (DOCA)-salt rats, a low renin model of hypertension in which ET-1 is overexpressed (123, 124). These effects of quercetin were associated with a reduction in both vascular and systemic oxidative stress (124). Quercetin protective effects against eNOS uncoupling were even maintained under glucotoxic conditions (131).

A RCT studied the acute effects of administering 200 mg of quercetin in 12 healthy men (136). Blood and urine samples taken, respectively, 2 and 5 h after oral ingestion of quercetin revealed a significant acute reduction in both the plasma and urinary concentrations of ET-1, translated into a reduced ET-1 production (136). This effect is substantial given the relatively small dose used and the low bioavailability of quercetin. Interestingly, it was reported that NO inhibits ET-1 production through the suppression of NFκB (182). A second mechanism involves the renin–angiotensin system: ACE inhibitors neutralize ACE by binding a zinc atom at the active site of the enzyme, which slows conversion of angiotensin I to angiotensin II, a powerful vasoconstrictor (183), including in human coronary arteries (184). Quercetin can chelate metal ions, including zinc (185), and it is tempting to presume it can act as an ACE inhibitor. However, available results have been discordant. *In vitro*, quercetin inhibited ACE activity in a concentration-dependent manner, with an IC_50_ of 310 μM (186). This value was significantly higher than that of captopril (0.02 μM) (186). In Wistar rats receiving an angiotensin-1 infusion, Hackl et al. showed that an attenuation of the BP was obtained with both oral and intravenous administration of quercetin (133). They also reported a 31% reduction in ACE activity in the quercetin group compared to the control group (133). In contrast, one double-blind placebo-controlled RCT did not find ACE activity inhibition after a single-dose of quercetin (187). In this study, five normotensive men and twelve hypertensive men ingested a total of 1,095 mg quercetin and 10 h later, the mean BP was reduced among the hypertensive patients by 5 mm Hg compared to the placebo group (187). Plasma ACE activity, ET-1, and brachial artery flow-mediated dilation were unaffected by quercetin, suggesting that the reduction in BP in hypertensive men was independent of the changes in ACE and ET-1 activity, or NO bioavailability (187).

Serban et al. conducted a systematic review of 7 RCTs published between 1998 and 2014, looking at the effects of quercetin on BP (137). Their meta-analysis revealed a significant reduction in systemic BPs associated with oral supplementation of quercetin (137). The weighed mean differences for the systolic and diastolic BPs were 3.04 mm Hg (*p* = 0.028) and 2.63 mm Hg (*p* < 0.001), respectively (137). These values are appreciable considering that the cohorts were largely made up of normotensive subjects. The doses of quercetin ranged from 100 to 1,000 mg/day. Interestingly, when using a meta-regression analysis, the systolic BP-lowering effect was only associated with the duration of supplementation, and not the administered dose, contrarily to the diastolic BP-lowering effects (137). Furthermore, when the RCTs were stratified according to the duration of supplementation, quercetin had no significant benefit in the subsets of studies lasting <8 weeks. Likewise, the BP values did not differ significantly between the two treatment arms in the subset of trials administering doses <500 mg/day. Altogether, these results indicate a significant anti-hypertensive effect of quercetin supplementation only when doses ≥500 mg/day are taken for ≥8weeks (137). Another meta-analysis which included 896 participants across 17 RCTS mirrored the results obtained by Serban et al., which indeed is the meta-analysis done by Huang et al. (138). More recently, a meta-analysis of 8 RCTs conducted among patients with metabolic syndrome traits showed that quercetin supplementation significantly reduced systolic BP, yet did not affect diastolic BP (139). Clearly, trials directly comparing different doses and regimen durations are needed.

### Senolytic Properties of Quercetin

The accumulation of SCs in the aging and diseased vessel wall raises the possibility that reducing senescence might delay deterioration of vascular structure and function. In an elegant and seminal experiment, Baker et al. demonstrated that the health span in progeroid mice can be enhanced by killing SCs using a transgenic suicide gene (188). Elimination of SCs also delayed progression of multiple age-related phenotypes, such as cancer, cataract, sarcopenia, lordokyphosis, loss of adipose tissue and skeletal muscle fibers, as well as improved exercise capacity (188). Translating that same effect into a druggable compound sparked research interest and led to the recent concept of “senolytic therapy” (140). Formed by the words “senescence” and “lytic” (destroying), a senolytic represents a molecule that could specifically induce cell death in SCs (189). Based on the knowledge that SCs survive despite their harsh internal state, the hypothesis was that this would be achieved by targeting their survival pathways and anti-apoptotic mechanisms (189). An alternative strategy to interfere with senescence would be to reduce the burden of SASP. The advent of antibody-based techniques such as sandwich enzyme-linked immunosorbent assay, and large-scale molecular biology techniques such as mRNA profiling, antibody arrays, proteomics or multiplex assays have made the detection and measurement of several SASP factors possible (190). These powerful tools therefore serve to test pharmaceutical efficacy of drugs that target SASP (190). In the following sections, we will see evidence suggesting that quercetin eliminates SCs and reduces the SASP.

In 2008, quercetin was reported to increase longevity of worms (191), but it was not until 2015 that its potential as a senolytic was highlighted in Kirkland's laboratory (140) (Table 1). First, the investigators identified a series of senolytic transcripts on pre-adipocytes. These included components of the ephrin regulating system, ephrin ligands B (EFNB), as well as the plasminogen-activated inhibitor-1 (PAI-1) and a member of the phosphatidylinositol-4,5-bisphosphate 3 kinase (PI_3_K) family, involved in regulating multiple cellular functions including survival (140). Then, they tested whether drugs that target any of these gene products would effectively induce apoptosis in radiation-induced senescent human pre-adipocytes and HUVECs. Of the 46 agents tested, quercetin and dasatinib, a non-specific tyrosine kinase inhibitor used for cancer therapy, were noticeably promising (140). Dasatinib is known to block EFNB-dependent suppression of apoptosis, while quercetin inhibits PI_3_K, other kinases and PAI-1, from the SERPIN family member (140, 192). In contrast to dasatinib, which was more effective on pre-adipocytes, quercetin preferentially reduced viability of senescent HUVECs (140). Parallel cultures of non-senescent HUVECs proliferated 2- to 3-fold in the presence of quercetin over the same period of 3 days, indicating that quercetin's induction of apoptosis is selective to SCs (140). In addition, the combination of dasatinib and quercetin achieved a synergistic effect by selectively killing both senescent pre-adipocytes and HUVECs, whom viability was, respectively, reduced by ~70% and ~50% (140). This suggests that using a mix of senolytics to target a broader range of anti-apoptotic networks may be a strategy to follow in developing future senolytic therapies (189). Used *in vivo*, the senolytic cocktail also reduced the number of p16-positive SCs in fat and liver from old mice (140). After a single 5 day treatment course of dasatinib+quercetin, the rodents exhibited an improved left ventricular ejection fraction (LVEF) and fractional shortening with no alteration of cardiac mass, as well as increased smooth muscle vascular reactivity to nitroprusside (140). Similar results were obtained by Xu et al.: 20 month-old mice who were fed dasatinib+quercetin intermittently for 4 months performed better at physical endurance tests compared to the control group (43). Next, they administered biweekly oral doses of dasatinib+quercetin to 24 to 27 month-old mice, equivalent to a human age of 75–90 years; compared to the controls, these mice had a 36% higher median post-treatment lifespan and a 65% lower mortality hazard (43). This was neither associated with an increased physical morbidity nor an increased age-related disease burden (43). In addition, in ApoE^−/−^ mice fed with a high-fat diet, dasatinib+quercetin given once monthly for 3 months was shown to decrease aortic calcifications and increase vascular reactivity (193). When used alone, quercetin increased the density of sirtuin 1 (Sirt1) in aorta of ApoE^−/−^ mice (141). Sirt1 functions as a nicotinamide adenosine dinucleotide (NAD+)-dependent deacetylase and is involved in genomic stability, basal level autophagy and cell survival (194). Sirt1 was found to delay both replicative and stress-induced senescence (194).

Hwang et al. conducted *in vitro* experiments with adult human coronary artery endothelial cells (HCAEC) from three deceased female donors using replicative senescence as a relevant model for human arterial aging (195). Contrary to the previous results reported in HUVECs (140), their findings showed that quercetin induces death in both early (non-senescent) and late-passage (senescent) HCAECs, without any selectivity for the latter (195). Quercetin's cytotoxicity was evident in all three donors at a concentration of 10 μM, which was half the amount used with HUVECs (140). Late-passage cells were more sensitive to quercetin's toxic effects as their relative cell abundance was already significantly decreased at a concentration of 6 μM (195). Their study also investigated hyperoside, also known as quercetin 3-D-galactoside, as an alternative to quercetin (195). Hyperoside is a natural derivative of quercetin produced by St. John's Wort and structurally identical except for a galactoside group attached in position 3 (Figure 2) (195). In contrast to quercetin, hyperoside had no significant cytotoxicity to either proliferating or late-passage HCAECs but was unable to display any senolytic activity (195). A second *in vitro* model of an adult human vasculature model was investigated by Jiang et al. using human aortic endothelial cells (HAECs) (141). In their study, senescence was induced by ox-LDLs. Their results revealed that quercetin decreased the expression of senescence-associated β-galactosidase and improved cell morphology of HAECs (141). The senolytic effect was dose dependent, as 0.3, 1, and 3 μmol/L of quercetin improved cells viability by 10.8, 40.9, and 48.9%, respectively (141). Quercetin simultaneously decreased ROS generation, also in a concentration-dependent manner. In addition, transcriptome microarray assays were performed and identified differentially expressed genes in the mRNAs profile of senescent HAECs treated with quercetin (141). Among them, several were involved in p53 and mammalian target of rapamycin (mTOR) signaling pathways, NO metabolism, maintenance of the cytoskeleton, extracellular matrix-receptor interaction, as well as complement and coagulation cascades, suggesting the potential mechanisms by which quercetin was effective against ox-LDLs (141). Quercetin also decreased the genetic expression of *AATK, CDKN2A*, and *IGFBP3* (141). *AATK* is induced during apoptosis, while *CDKN2A* (p16) is one of the most important senescence markers (141). Interestingly, a high circulating concentration of *IGFBP3* was found to be a predictor of IHD (196). One can therefore wonder if quercetin could alleviate the risks of IHD in patients by decreasing *IGFBP3*.

Clinical trials studying the senolytic effects of quercetin remain scarce (Table 1). In the first clinical trial of senolytics, an intermittent regimen of dasatinib+quercetin (dasatinib: 100 mg/day, quercetin: 1,250 mg/day, 3 days/week over 3 weeks) improved physical tolerance, but not pulmonary function, in patients with idiopathic pulmonary fibrosis, a fatal senescence-associated disease (197). Another open label pilot study was conducted by Hickson et al. in 9 adults aged 50–80 years with diabetic kidney disease (142). The patients received a 3 day oral treatment regimen with dasatinib 100 mg daily and quercetin 500 mg *bid*. Eleven days after treatment completion, there was a significant reduction in the number of adipose tissue SCs and circulating SASP factors, including IL-1α, IL-6, MMP-9, and MMP-12, accompanied by an increase of adipocyte progenitors, suggesting a selective cytotoxic effect for SCs (142). These results are in agreement with a previous *in vitro* study performed on human omental tissue resected during gastric bypass surgery (43). The surgical explants treated with a dasatinib+quercetin medium for 48 h had significantly less SCs and a lower secretion of SASP components compared to the explants treated with a vehicle (43). To the best of our knowledge, no clinical trial has yet examined the senolytic effects of quercetin on endothelial dysfunction in humans, in the context of CVD.

### Myocardial Protective Effects of Quercetin

The beneficial effects of quercetin on dyslipidemia, hypertension, senescence and other risk factors can be seen as a primary prevention measure against endothelial dysfunction. Once the endothalial dysfunction has resulted in an adverse cardiac event, secondary and tertiary prevention strategies become crucial in order to reduce the progression of the disease and its impacts on patients' quality of life. One of the most striking examples is myocardial ischemia. In the latter, dysfunctional endothelial cells of the coronary arteries induce a local disturbance in other cell lines, including cardiomyocytes and fibroblasts (198). They trigger a host response which includes increased oxidative stress, calcium imbalance, as well as cytokine, platelets, and leukocytes activation (198). This endothelial dysfunction is also a critical mediator of myocardial dysfunction after reperfusion (198). In response, many pathological adaptations occur, such as increased extracellular matrix deposition leading to myocardial interstitial fibrosis, changes in the myocardial cell morphology, and eventually, ventricular dilatation (199). The latter, called “ventricular remodeling” is detrimental to ventricular compliance and contractility (199). Clinically, it translates into debilitating conditions, ranging from stable angina to myocardial infarction and heart failure. In addition, experimental data showed that endothelial dysfunction correlates with the degree of myocardial injury, both from the ischemic and reperfusion insults (198). These observations suggest that quercetin's ability to minimize myocardial injury following an ischemic event may be, at least partly, mediated by its effects on the endothelium.

Many *in vivo* and *ex vivo* murine studies have shown both functional and structural benefits of exposing myocardium to quercetin in an acute ischemic setting (143–153) (Table 1). These studies used rodent models in which transient myocardial injury was induced by ISO injections, surgical occlusion of the left coronary artery (LAD) or interruption of Langendorff perfusion (123, 124, 130, 131, 134–136, 178–181). Quercetin was either given as an oral gavage, an intravenous or intraperitoneal infusion (123, 124, 130, 131, 134–136, 178–181). Measured functional hemodynamic parameters included left ventricular end-diastolic pressure (LVEDP), left ventricular systolic pressure (LVSP) and maximal ratio of pressure change during isovolumetric contraction (peak dP/dt). While myocardial ischemia systematically decreased LVSP and peak dP/dt, and increased LVEDP, this effect was counteracted by quercetin (143–147). Liu et al. used echocardiography in mice to estimate left ventricular function (148). They showed that quercetin significantly slowed the decline in LVEF and fractional shortening compared with the control group (148). On macroscopic examination, treatment with quercetin induced a significant reduction of myocardial infarct size on triphenyl tetrazolium chloride (TTC) staining (149–151). This was further supported by lower levels of serum creatine kinase (CK), CK-MB, cardiac troponin T and lactate dehydrogenase, all enzymatic markers of myocardial insult (144, 147–149, 151–155). Histopathological examinations also revealed lower infiltration of leukocytes to the site of infarction, less edema and overall maintained tissue architecture (149, 152, 153). All these findings are in favor of a preservation of cardiomyocytes' membrane integrity and global improvement in myocardial function after exposure with quercetin. Interestingly, these cardioprotective effects were observed whether quercetin was administered before induction of ischemia or during reperfusion. This suggests that quercetin may have both ischemic preconditioning and postconditioning capacities.

**Figure 4:**
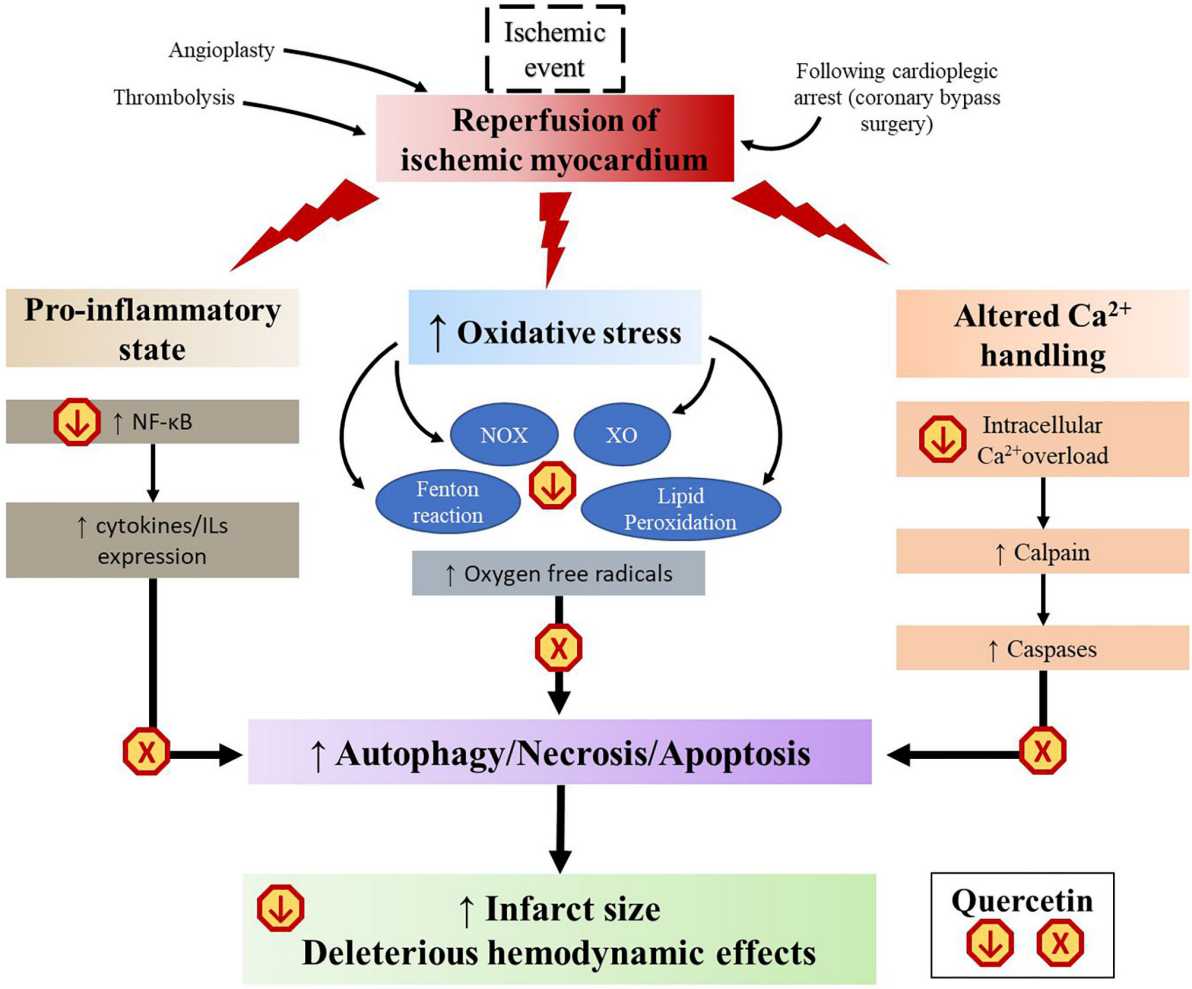
Schematic representation of the multistep mechanisms of quercetin to mitigate myocardial ischemic reperfusion injury. XO, xanthine oxidase; NOX, NADPH oxidase.

Although timely reperfusion is essential for myocardial salvage, it is accompanied by a stress reaction known as “myocardial ischemia-reperfusion injury” (MIRI), which paradoxically increases the degree of myocardial damage (200). As restoration of the circulation allows blood to reach cells that were previously subjected to ischemia, sudden availibility of oxygen leads to a burst in the generation of ROS, mainly deriving from the Fenton reaction, NOX, and xanthine oxidase (XO) (200). These redox reactions lead to formation of oxygen radicals, lipid peroxidation, calcium overload, activation of inflammatory cascades, and apoptosis, which propagate and cause myocardial damage even distant to the original site of insult (200). This has important clinical implications as it limits the benefits of current revascularization therapies such as thrombolysis, angioplasty or coronary artery bypass surgery (200). A number of studies based on the rodent models of transient myocardial infarction have suggested that quercetin attenuates MIRI by interfering with several of these pathways (Figure 4).

First, quercetin has well-documented antioxidant properties. Thanks to its chemical structure (Figure 2), it is able to directly scavenge free radicals such as superoxide, hydrogen peroxide, peroxyl, and hydroxyl radicals (45). Quercetin can also reduce the formation of ROS by inhibiting NOX and XO, decreasing the activity of cyclooxygenase and LOX, as well as regulating the activity of intracellular signaling cascades involved in inflammatory reactions (53). Chemical studies revealed that quercetin can reversibly inhibit XO-catalyzed uric acid and superoxide radicals formation in a double-displacement reaction (201, 202). However, results of *in vivo* studies remain controversial (203). In a hyperuricemic mouse model, quercetin given orally at 100 mg/kg for 1 to 7 consecutive days reduced serum urate levels and XO hepatic activity in a concentration-dependent manner (204). In another study using the same model, a 7 day treatment of 400 mg/kg orally administered quercetin failed to reduce both serum urate levels and XO expression (205). In a rabbit model of surgically-induced MIRI, an intravenous injection of quercetin given 5 min before ligation attenuated the enzymatic activity of NOX2 expressed in endothelial cells (206). On the other hand, quercetin acts as a chelating agent. It can inhibit the Fenton reaction by interfering with ferrous iron (207). It can also bind to zinc and facilitate zinc trafficking into cells (208), which in turn functions as an antioxidant (209). Lipid peroxidation is the process by which unsaturated fatty acids are converted to lipid peroxyl radicals by hydrogen oxidation, which, in turn, extract hydrogen from other fatty acid molecules to create more free radicals (210). Some studies reported that quercetin offers a protection against lipid peroxidation chain reaction by neutralizing peroxyl radicals and by binding to transition metal ions, catalyzers of lipid peroxidation (154, 211, 212). Finally, quercetin pretreatment was shown to decrease the content of malondialdehyde (MDA), a mutagenic product of lipid peroxidation chain reaction, and to potentiate the activity of superoxide dismutase (SOD) and glutathione peroxidase (GSH-Px), two most important antioxidases in cardiomyocytes (144, 147, 148, 152, 213, 214). All these properties allow quercetin to slow down the domino effects of free radical injury in MIRI.

With myocardial ischemia and MIRI, there is a shift toward a pro-inflammatory and pro-apoptotic phenotype caused by an increased secretion of cytokines (200). As seen previously, quercetin was shown to significantly repress this inflammatory cascade, both *in vivo* and *in vitro* (95). The pro-inflammatory response is further exacerbated by activation of NFκB, which is a pivot transcription factor in promoting cytokine expression. Enhanced NFκB signaling induces a positive feedback, which further prompts inflammasome assembly (35). NFκB can be activated through the interaction of high mobility group box-1 (HMGB1) with toll-like receptors (TLRs) located in cardiomyocytes (215). HMGB1 has been found to be released by necrotic cardiomyocytes under ischemic conditions and may serve as an early mediator of inflammation following MIRI (215). Western blot analyses revealed a strong activation of the HMGB1/TLR/NFκB pathway in heart tissues after ischemic/reperfusion stimulation in LAD ligated rats (151). Treatment with quercetin significantly inhibited expression of HMGB1 and TLR4 (151, 153). In addition, up-regulation by quercetin of peroxisome proliferation-activated receptor gamma (PPAR-γ) further supports the targetting of NFκB activation (148). Several reports revealed that PPAR-γ, a ligand-activated nuclear transcription factor, could suppress the signal transduction of NFκB pathway in vascular diseases (216). A study demonstrated that mice with transient LAD ligation that received a 10 day pre-treatment of quercetin had a significantly higher number of PPAR-γ positive myocardial cells (148). The authors also found that quercetin partially reversed the effects of a PPAR-γ inhibitor, GW9662, compared to non-quercetin-treated mice, with an associated improvement in LVEF, fractional shortening and cardiac biomarkers (148). Lastly, quercetin was shown to protect against calcium overload. Elevated intracellular Ca^2+^ is involved in the deleterious biochemical and functional changes accompanying MIRI (217). *In vitro*, quercetin decreased Ca^2+^-dependent cell death when added to H9C2 cardiomyocyte 30 min before application of H_2_O_2_-induced oxidative stress (218). Furthermore, the downstream Ca^2+^ activated calpain pathways may lead to contractile dysfunction and cytoskeleton damage (219). Increased calpain activity has been reported as an aggravating factor in myocardial infarction (219). Oral quercetin (50 mg/kg) pre-treatment of Wistar rats exposed to an ISO-induced myocardial infarction downregulated the genetic expression of calpain 1 and 2, protecting the myocardium from their overactivity (152). This cardioprotective effect was also supported by a reduction of CK-MB and cardiac troponin T in quercetin-treated rats compared to the control group (152). Another *in vivo* study found that quercetin prevented inhibition of the sodium-potassium and the calcium pumps caused by myocardial infarction (149).

Despite extensive experimental data suggesting that quercetin can attenuate MIRI, very few trials have explored the use of quercetin for the treatment of myocardial ischemia in humans. In the study done by Chekalina et al., 30 out of 85 patients with stable angina on optimal medical therapy were given quercetin at a daily dose of 120 mg for 2 months (156). The quercetin patients had lower levels of IL-1β and TNF-α compared to the control group (156). An open-label clinical trial conducted in Ukraine studied the administration of intravenous quercetin (Corvitin) over 10 days in patient admitted with an acute myocardial infarction and heart failure symptoms. After 3 days, there was a significant improvement in their profile of cardiac biomarkers and LVEF (157). Altogether, these clinical data suggest that quercetin has potential cardioprotective effects, and form a solid foundation for a potential application of quercetin in the prevention of IHD and its complications. This hypothesis remains to be tested in large clinical trials.

## Endothelial-to-Mesenchymal Transition: a Less Explored Player in Endothelial Dysfunction

In the late 1990's, endothelial cells were found to undergo a highly dynamic process of dedifferentiation known as endothelial-to-mesenchymal transition (EndoMT) (220). During EndoMT, endothelial cells progressively acquire a wide spectrum of phenotypes characteristic of multipotent cells (221). This phenotype switch involves a reduced expression of distinctive endothelial cells markers such as von Willebrand factor, vascular endothelial-cadherin or CD31/PECAM-1 and an increased expression of mesenchymal cells markers such as alpha-smooth muscle actin, vimentin and N-cadherin (220, 222). Similar to senescence, EndoMT is a double-edged sword. EndoMT-derived cells exhibit hallmarks of invasive cells through cytoskeletal reorganization, increased ECM production, loss of cellular adhesion and resultant enhanced migratory potential (223, 224). This process is critical in the developing embryo where it was shown to generate vasculogenesis and the cardiac cushions for valve development (225). EndoMT was also shown to contribute to wound healing (226). However, when triggered under certain pathological conditions such as inflammation or shear-stress injuries, EndoMT can give rise to cancer progression (224), fibrodysplasia ossificans progressive (221), pulmonary arterial hypertension (227, 228) or cardiac and renal fibrosis (229, 230). The best-studied mediator of EndoMT is TGF-β (231). The latter can induce EndoMT either directly, through both *Smad*-dependent and *Smad*-independent pathways (232, 233), or indirectly, through ET-1 (234, 235), CAV-1 (236), or NFκB (151, 153, 237).

Strong lines of evidence support the cross-links between endothelial dysfunction, atherosclerosis, hypertension, senescence and EndoMT (228, 238–241). Targeting EndoMT opens therefore a new therapeutic avenue against CVD. However, contrarily to the other players of endothelial dysfunction, few studies specifically looked at the potential contribution of quercetin. In their study performed *in vitro*, Huang et al. showed that quercetin effectively inhibited TGF-β1-induced human pulmonary arterial endothelial cells proliferation and transdifferentiation (242). This suggests that quercetin may be a potential antagonist for a pathogenic model of pulmonary artery hypertension secondary to pulmonary arterial endothelial cells excessive growth. Moreover, as discussed in previous sections, experiments done outside the scope of EndoMT have demonstrated that quercetin can downregulate ET-1 (134, 135), CAV-1 (118), and NFκB, which are all mediators of EndoMT. Other studies explored the effects of quercetin on a similar process as EndoMT but involving epithelial cells, hence the name “epithelial-to-mesenchymal transition” (EMT). In human retinal pigment epithelial cells (ARPE-19) exposed to TGF-β1, quercetin suppressed proliferation, migration, and collagen I secretion (243). It also downregulated EMT-related markers such as alpha-smooth muscle actin and N-cadherin; conversely, it upregulated the expression of tight junction proteins and epithelial-cadherin (243). In addition, quercetin inhibited Smad2/3 phosphorylation and translocation of Smad4, suggesting that the progression of EMT in ARPE-19 cells was reversed *via* the Smad pathway (243). Incubation with quercetin also reduced EMT in mammary carcinoma and prostate cancer cell lines (244, 245). Together, these results support a role for quercetin against EndoMT and EMT. However, despite multiple similarities between the two processes, including canonical TGF-β signaling as their driving force (246), more research in EndoMT models is needed to further confirm the efficacy of quercetin in targeting its trigger mechanisms.

## Discussion

**Figure 5:**
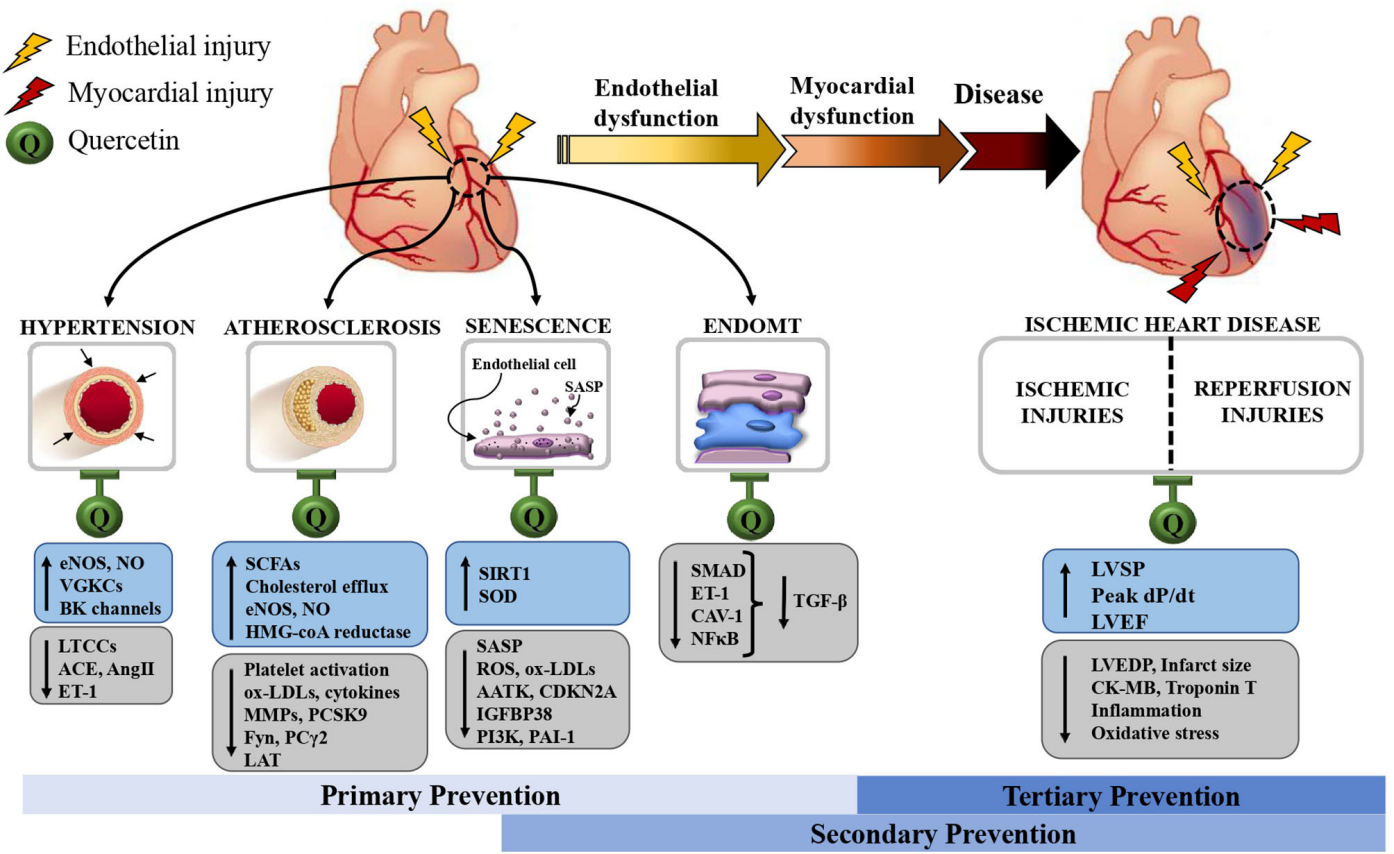
Schematic representation of the endothelial and, by extension, myocardial protective effects of quercetin. These allow quercetin to act as a primary, secondary and tertiary preventive measure against cardiovascular diseases. AATK: apoptosis-associated tyrosine kinase; ACE: angiotensin-converting enzyme; AngII, angiotensin II; BK, big K, large-conductance Ca^2+^-sensitive K^+^ channels; CAV-1, caveolin-1; CDKN2A, p16, cyclin-dependent kinase inhibitor 2A; CK-MB, creatinine kinase-MB; EndoMT, endothelial-to-mesenchymal transition; ET-1, endothelin-1; IGFBP3, insulin-like growth factor binding protein-3; eNOS, endothelial nitric oxide synthase; NFκB, nuclear factor-kappa B; NO, nitric oxide; ox-LDLs, oxidized low density lipoproteins; Fyn, Src family 59 kDa non-receptor protein tyrosine-kinase; LAT, linker for activation of T cells; LTCCs, L-type Ca^2+^ channels; LVEDP, left ventricular end-diastolic pressure; LVEF, left ventricular ejection fraction; LVSP, left ventricular systolic pressure; MMPs, matrix metalloproteases; PAI-1, plasminogen-activated inhibitor-1; PCSK9, proprotein convertase subtilisin/kexin type 9; PI_3_K, phosphatidylinositol-4,5-bisphosphate 3 kinase; PLCγ2, phospholipase Cγ2; SCFAs, short-chain fatty acids; ROS, reactive oxygen species; SASP, senescence-associated secretory phenotype; SIRT1, sirtuin-1, nicotinamide adenine dinucleotide [NAD(+)]-dependent protein deacetylase; SOD, superoxide dismutase; TGF-β, transforming growth factor beta; VGKCs, voltage-gated K^+^ channels.

The magnitude of the role of endothelial dysfunction in CVD is well-established. Many pathophysiological processes are involved, and they contribute to each other in a feedback manner, as seen with the triad of vascular senescence, hypertension and atherosclerosis. This also means that each pathway is a potential target for alleviating endothelial dysfunction. Numerous drugs are already available to effectively treat dyslipidemia and hypertension. In comparison, anti-senescence therapy is only a nascent yet promising research field. Development of senolytic drugs would bring a conceptual change that an aging vessel is not an immutable process. On the other hand, an important clinical consequence of endothelial dysfunction is manifested in IHD. The burden of myocardial ischemia has been improved with more timely and effective reperfusion strategies such as angioplasty, bypass surgery, antiplatelet, and antithrombotic agents used to restore the patency of infarct-related coronary arteries. However, at present, there is still no effective therapy to prevent MIRI. In this review, we have described mechanistic, experimental and clinical evidence that suggests quercetin can manifest a wide range of cardioprotective biological activities (Table 1). Not only does it have anti-hypertensive and anti-atherosclerotic effects, but it also seems to mitigate senescence and MIRI, two Achilles' heels in the modern treatment of CVD. Moreover, although still scarce, encouraging data suggest that quercetin may also act against abnormal EndoMT, an important yet less explored player in endothelial dysfunction. These properties of quercetin form the basis for its potential benefits against aging-related endothelial dysfunction and CVD (Figure 5).

While this review has focused on conditions originating from a diseased endothelial layer, prevention of endothelial dysfunction can be achieved by intervening beyond the endothelium itself. Indeed, endothelial dysfunction is undeniably associated with the remaining entities of the metabolic syndrome: obesity and insulin resistance (247–251). Their role as independent risk factors for CVD merits as much attention as hypertension and dyslipidemia (252). In fact, the increasing incidence of obesity and corresponding rise in type-2 diabetes are further challenging the prevention and treatment of CVD (253). The metabolic effects of quercetin against these two conditions have been equally encouraging and were recently reviewed elsewhere (254–258). In addition, it should be emphasized that, for clarity reasons, the effects of quercetin on the various biochemical and biomechanical signaling cascades involved in endothelial dysfunction were discussed as separate entities. However, in reality, senescence, vasomotor dysfunction, atherosclerosis, EndoMT, inflammation, oxidative stress, and altered endothelial cellular metabolism all interact, cross talk and occur simultaneously. The resulting chain reactions create a vicious circle, which, once it is established in one person, can easily multiply their cardiovascular risks. The ability of quercetin to act as such a versatile multi-target agent against the domino effect of endothelial dysfunction becomes very appealing. It seems to be promising not only in primary prevention, but also in secondary and tertiary prevention against the dysfunctional coronary endothelium and myocardium exposed to ischemia-reperfusion injuries.

However, after being studied for two decades and showing encouraging *in vitro* and *in vivo* results, quercetin still occupies a modest title as a dietary supplement. This could be explained by a couple of factors. First, quercetin lacks molecular specificity. It does not radically block a metabolic pathway nor inhibit a receptor of interest. Instead, quercetin has a wide variety of biological activities, which makes it difficult to establish a clear association between its administration and the observed positive effects. As a matter of fact, quercetin's role as a direct senolytic agent is still open for discussion. Does it selectively target SCs, or does it improve their clearance by off-target mechanisms such as antioxidant activity? Second, clinical trials using more consistent protocols are needed to consolidate the medical findings attributed to quercetin. Published trials have been using various treatment durations, doses and routes of administration, certainly contributing to heterogenous findings. The considerable variation in bioavailability of quercetin among individuals might result in subtherapeutic plasma concentrations, especially when using lower doses. Furthermore, *in vitro* experiments most often administered a hit-and-run treatment with quercetin. We could hypothesize that replicating this regimen in humans would yield more substantial effects. Lastly, to quote Samuel Butler, “*medicine is not practiced as an art of drawing sufficient conclusions from insufficient premises*.” In the case of quercetin, the absence of its medical use despite its appealing properties might suggest that we should reconsider our expectations for its potential therapeutic implications. If a defect of the endothelium was accountable for a disease, and a drug could be given which would correct the defect, the disease would obviously be cured. However, drugs that were to act through less direct principles might still be useful. For example, if quercetin's well-established anti-inflammatory and antioxidant effects, albeit less specific, could make the endothelium less vulnerable to injury and senescence, it could potentially reinforce the efficacy of other cardiovascular agents. Ultimately, managing CVD has never been about carrying a hammer and treating everything “as if it were a nail.” Instead, it revolves around adding different tools to tackle different issues of a large-scale problem.

In conclusion, quercetin represents a promising natural compound that appears to satisfy all the requirements to develop a nutraceutical against endothelial dysfunction. There is a pressing need for well-designed clinical trials that explore its intriguing potential for senolytic therapy and myocardial protection.

## Author Contributions

OD, NT-T, and ET designed the project and its main conceptual ideas. OD performed the research strategy, data collection, data analysis and interpretation, drafted the manuscript, and designed the figures, with input from all authors. All authors provided critical revisions to the article and approved the final version for publication.

## Conflict of Interest

The authors declare that the research was conducted in the absence of any commercial or financial relationships that could be construed as a potential conflict of interest.
